# Identification and functional analysis of senescent cells in the cardiovascular system using omics approaches

**DOI:** 10.1152/ajpheart.00352.2023

**Published:** 2023-09-01

**Authors:** Sophia A. Mahoney, Amit K. Dey, Nathan Basisty, Allison B. Herman

**Affiliations:** ^1^Department of Integrative Physiology, University of Colorado at Boulder, Boulder, Colorado, United States; ^2^Intramural Research Program, National Institute on Aging, National Institutes of Health, Baltimore, Maryland, United States

**Keywords:** aging, biomarkers, cellular senescence, proteomics, transcriptomics

## Abstract

Cardiovascular disease (CVD) is a leading cause of morbidity and mortality worldwide, and senescent cells have emerged as key contributors to its pathogenesis. Senescent cells exhibit cell cycle arrest and secrete a range of proinflammatory factors, termed the senescence-associated secretory phenotype (SASP), which promotes tissue dysfunction and exacerbates CVD progression. Omics technologies, specifically transcriptomics and proteomics, offer powerful tools to uncover and define the molecular signatures of senescent cells in cardiovascular tissue. By analyzing the comprehensive molecular profiles of senescent cells, omics approaches can identify specific genetic alterations, gene expression patterns, protein abundances, and metabolite levels associated with senescence in CVD. These omics-based discoveries provide insights into the mechanisms underlying senescence-induced cardiovascular damage, facilitating the development of novel diagnostic biomarkers and therapeutic targets. Furthermore, integration of multiple omics data sets enables a systems-level understanding of senescence in CVD, paving the way for precision medicine approaches to prevent or treat cardiovascular aging and its associated complications.

## INTRODUCTION

Cellular senescence is a complex biological process characterized by the irreversible growth arrest of cells in response to various stimuli, including DNA damage, telomere shortening, oxidative stress, and oncogene activation (1, 2). Senescent cells exhibit a distinct phenotype, including enlarged and flattened morphology, increased expression of senescence-associated β-galactosidase (SA-β-Gal), and secretion of a wide range of proinflammatory cytokines, chemokines, and growth factors collectively referred to as the senescence-associated secretory phenotype (SASP) (2, 3).

Although cellular senescence plays a critical role in many physiological processes, including embryonic development, wound healing, and tumor suppression, the accumulation of senescent cells has been implicated in various age-related pathologies, including cardiovascular disease (CVD) (4). CVD is the leading cause of morbidity and mortality worldwide and encompasses a wide range of conditions, including atherosclerosis, heart failure, myocardial infarction, and stroke (5). Atherosclerosis, in particular, is a chronic inflammatory disease characterized by the accumulation of lipid-rich plaques within the arterial walls. The progression of atherosclerosis involves the recruitment of inflammatory cells, including monocytes, T cells, and mast cells, to the arterial intima, where they interact with endothelial cells (ECs) and vascular smooth muscle cells (VSMCs). These cells become activated, producing reactive oxygen species (ROS), cytokines, and chemokines, which promote the recruitment of additional inflammatory cells and the migration and proliferation of VSMCs, ultimately leading to the formation of atherosclerotic lesions (6).

Recent evidence suggests that cellular senescence plays a crucial role in the development and progression of atherosclerosis. Senescent cells have been identified within atherosclerotic plaques in both animal models and human patients, and senescence-associated markers, including p16 (CDKN2A), IL-6 (IL6), and SA-β-Gal, have been detected in vascular cells in atherosclerotic lesions (7–9). Furthermore, SASP factors have been shown to promote the recruitment and activation of immune cells, including monocytes, and to increase the migration and proliferation of VSMCs (10). The accumulation of senescent cells in the arterial walls may contribute to the chronic inflammation and oxidative stress, characteristic of atherosclerosis, leading to the destabilization of plaques and the development of acute cardiovascular events, such as myocardial infarction and stroke (11, 12). Senescent cells may also contribute to the development of other cardiovascular conditions, including heart failure, by promoting fibrosis and impairing cardiac function (13, 14).

**Figure 1.**
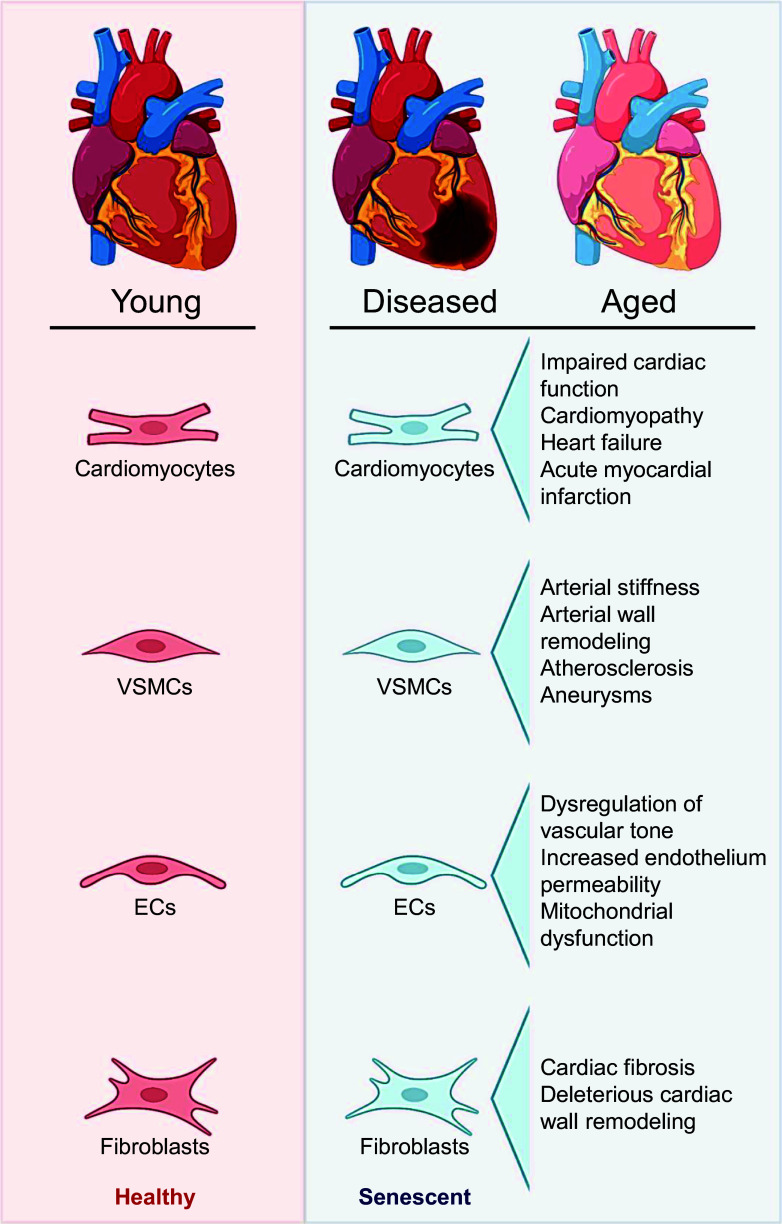
Overview of cardiovascular cell senescence. Cells originating from the heart and vascular tissue are prone to enduring nonlethal stressors, particularly during disease or aging, leading to the development of a phenotypic state referred to as cellular senescence. Consequently, senescent cardiomyocytes, vascular smooth muscle cells (VSMCs), endothelial cells (ECs), and fibroblasts have all been implicated in the pathogenesis of diverse cardiovascular-related pathologies. The detrimental consequences of senescent cells in cardiovascular diseases (CVDs) and aging have sparked considerable interest in the development of senescence-targeting drugs aimed at inhibiting or selectively eliminating these cells. Images were created with a licensed version of BioRender.com and Photoshop.

The identification and targeting of senescent cells in atherosclerosis and other cardiovascular diseases have emerged as a promising therapeutic strategy to improve cardiovascular health outcomes. Several approaches have been proposed, including the use of senolytic drugs, which selectively induce apoptosis in senescent cells, and senomorphics which suppress the SASP to reduce the burden of senescent cells (15). Senolytics uniquely take advantage of the enhanced prosurvival and antiapoptotic pathways expressed in senescent cells and promote apoptosis by targeting these critical proteins, such as p53 and the Bcl-2 family, among others (16). Senomorphics, on the other hand, suppress the SASP produced by senescent cells by targeting NF-κB, mTOR, and p38 MAPK pathways, and others. Importantly, because senescent cells are heterogeneous, some senotherapeutic compounds have both senolytic and senomorphic capabilities (16). Many of these senotherapies have been tested in animal models of cardiovascular disease and aging with highly promising outcomes (Table 1). Several challenges remain to generate clinically relevant senotherapies, including the identification of reliable senescence markers specific to vascular and cardiac cells and the potential off-target effects of senolytic drugs on nonsenescent cells. Undoubtedly, cellular senescence is an important contributor to the development and progression of CVDs, including atherosclerosis (Fig. 1). Further research using high-throughput, unbiased omics technologies would improve our understanding of the mechanisms driving senescence in vascular and cardiac cells, generating effective strategies for targeting senescent cells in the context of CVD. Such advances may lead to significant upgrades in the prevention and treatment of age-related cardiovascular diseases, ultimately improving the health outcomes of millions of individuals worldwide. In this review, we will highlight the role of senescent cells in CVDs and explain that a key path to unlocking their therapeutic potential lies in the use of omics-based strategies.

**Table 1.** Studies of senotherapeutics in animal models of cardiovascular disease and their outcomes

Model	Condition	Senolytic Treatment	Cellular Senescence Outcomes	Cardiovascular Outcomes	Reference
C57Bl/6 mice	Old age	D + Q	↓SA-β-Gal and *Cdkn2a* expression	Improved ejection fraction and endothelial function	(17)
C57Bl/6 and INK-ATTAC mice	Old age	ABT-263 and genetic	↓p16 abundance and DNA damage	Ameliorated myocardia hypertrophy and fibrosis	(18)
C57Bl/6 and INK-ATTAC mice	Old age	D + Q and genetic	↓p16 abundance, SA-β-Gal, and DNA damage	Cardiac progenitor cell activation and cardiomyocyte formation	(19)
C57Bl/6 mice	Doxorubicin-induced heart failure	ABT-263	↓p16, p21 and p53 expression and abundance	Improved ejection fraction and cardiac function	(20)
C57Bl/6 mice	Angiotensin II-induced heart failure	ABT-263	↓p16 and p21 abundance	Improved ejection fraction	(21)
C57Bl/6 mice	High-fat diet-induced heart failure	Q	No change in SA-β-Gal and ↑*Cdkn2a* and *p53* expression	Restored heart size and reduced fibrosis	(22)
C57Bl/6 mice	Ischemia-reperfusion injury in old mice	ABT-263	↓*Cdkn2a* expression	Increased survival following myocardial infarction	(23)
Fisher 344 rats	Ischemia-reperfusion injury	ABT-263	↓p16, p21, and SASP expression and abundance	Improved ejection fraction and cardiac function	(24)
C57Bl/6 mice	Myocardial infarction in old mice	D + Q	↓p16 abundance and DNA damage	Improved left ventricular and cardiac function	(25)
C57Bl/6 mice	Myocardial infarction with reperfusion	ABT-263	↓*Cdkn1a*, *Cdkn2a*, and SASP expression	Ameliorated myocardial hypertrophy and fibrosis and improved ejection fraction	(26)
p16-3MR mice	Myocardial infarction with reperfusion	Genetic	↓p16, p21, and SASP abundance	Improved ejection fraction and reduced fibrosis	(27)
C57Bl/6 and p16-3MR mice	Old age	ABT-263 and genetic	↓p16 abundance	Improved endothelial function and reduced arterial stiffness	(28)
C57Bl/6 and p16-3MR mice	Old age	Fisetin	↓p16 abundance	Improved endothelial function	(29)
C57Bl/6 mice	Old age and apoe KO-induced atherosclerosis	D + Q	↓DNA damage	Improved vasomotor function, reduced arterial stiffness, and ameliorated atherosclerotic plaque	(30)
C57Bl/6 mice	Old age and apoe KO-induced atherosclerosis	BPTES	↓SA-β-Gal and SASP abundance	Ameliorated atherosclerotic plaque	(31)
C57Bl/6 mice	Apoe KO-induced atherosclerosis	ABT-263	↓*Cdkn2a* expression and p21 abundance	Ameliorated atherosclerosclerotic plaque	(32)
p16-3MR mice	Apoe KO-induced atherosclerosis	ABT-263 and genetic	No change in *Cdkn2a* or SASP expression	Ameliorated atherosclerosclerotic plaque	(33)
C57Bl/6 mice	Apoe KO-induced atherosclerosis	GPNMB vaccine	↓*Cdkn1a* and *Cdkn2a* expression and SA-β-Gal	Ameliorated atherosclerosclerotic plaque	(34)
p16-3MR mice	Ldlr KO-induced atherosclerosis	ABT-263 and genetic	↓SA-β-Gal and *Cdkn2a* expression	Ameliorated atherosclerosclerotic plaque	(7)
C57Bl/6 and INK-ATTAC mice	Ldlr KO-induced atherosclerosis	ABT-263 and genetic	↓SA-β-Gal	Ameliorated atherosclerosclerotic plaque	(35)
C57Bl/6 mice	Old mice and angiotensin II-induced abdominal aortic aneurysm	D + Q	↓*Cdkn1a* and SASP expression	Ameliorated abdominal aortic aneurysm	(36)

D + Q, dasatinib and quercetin; Q, quercetin; BPTES, *bis*-2-(5-phenylacetamido-1,3,4-thiadiazol-2-*yl*)ethyl sulfide; GPNMB, glycoprotein nonmetastatic melanoma protein B; SA-β-Gal, senescence-associated β-galactosidase; SASP, senescence-associated secretory phenotype.

### Cardiac Senescence

Cellular senescence has been implicated in both beneficial and harmful capacities in the heart (37). Under healthy physiological conditions, evidence suggests that cellular senescence aids in heart regeneration and cardiac remodeling (38, 39). However, in states in which senescent cells accumulate, tissue damage is exacerbated by the toxic microenvironments created by senescent cells and the SASP (40). Increasing evidence suggests that cellular senescence contributes to cardiac pathologies such as heart failure (41), myocardial ischemia and infarction (23, 26, 38, 42), and cancer chemotherapy-related cardiotoxicity (43, 44). Thus, understanding the molecular and cellular changes that the heart undergoes because of the accumulation of senescent cells is of high biomedical importance.

Cardiomyocytes make up the majority of cells in the heart, as postmitotic cells typically undergo senescence because of intrinsic and extrinsic stressors, such as DNA damage, mitochondrial dysfunction, and oxidative stress, rather than replicative exhaustion (18, 45, 46). Early studies observed the presence of cellular senescence in aged hearts as characterized by changes in cardiomyocyte morphology, marked by increased cell volume, and reduced cell proliferation (47). Doxorubicin, a common anthracycline chemotherapeutic agent and established cellular senescence inducer, has also been shown to promote cardiomyocyte senescence, with cells displaying increased cell volume and flattened morphology (44). The molecular mechanisms of senescent cardiomyocytes have yet to be fully elucidated; however, emerging data suggest that senescent cardiomyocytes express canonical markers and regulators of senescent cells including the secretion of SASP factors, higher ROS production, and high expression levels of p53 (TP53) or p16 (46).

In addition to cardiomyocytes, cardiac fibroblasts also contribute to cardiac cell senescence (48). A major characteristic of cardiac fibroblasts is their production and secretion of growth factors, cytokines, extracellular matrix components, and other signaling molecules that maintain the cardiac tissue structure. Physiologically, early cardiac fibroblast senescence following myocardial infarction is thought to be protective against fibrosis by secreting SASP factors that recruit immune surveillance cells for tissue repair (48). However, excess senescent fibroblasts in the heart have been shown to be vital in cardiac fibrosis pathogenesis by promoting deleterious cardiac wall remodeling (49).

Aside from increased senescent fibroblasts and SASP, senescent cardiomyocytes are also thought to contribute to multiple clinical cardiac pathological disorders and have been associated with impaired cardiac function. Early clinical evidence found an association between cardiac levels of p53, cardiomyopathy incidence, and end-stage heart failure (50). Since then, several clinical studies have identified the presence of senescent cardiomyocytes in cardiomyopathy (51, 52), myocardial ischemic damage (38), and acute myocardial infarction (53). In these studies, markers of cellular senescence were increased in patients with heart disease compared with patients with healthy hearts. Despite the associations observed between cellular senescence and heart disease, studies investigating the direct role of senescent cells in cardiac dysfunction are limited given the challenges in obtaining clinical samples. Thus, several preclinical models have emerged to elucidate the mechanisms by which senescent cells contribute to cardiac diseases and dysfunction.

Cardiac function is impaired with increased chronological age and in conditions of accelerated aging (54). Impaired cardiac function is observed in vivo by reduced cardiac output, ejection fraction, left ventricular systolic and diastolic volume, stroke volume, as well as morphological alterations such as heart hypertrophy and fibrosis in mice. Similar to clinical observations, increased p53 expression in the hearts of mice is associated with cardiac dysfunction such as increased left ventricular weight, reduced ventricular function, and suppression of angiogenesis (55). Moreover, doxorubicin-treated rats, a model of accelerated cardiovascular aging, have increased cardiac cell senescence biomarkers and signs of cardiotoxicity including impaired cardiac ejection fraction and diastolic volume compared with control animals (44). Pharmacological clearance of senescent cells in old mice, using the well-established senolytic ABT-263, improves age-associated pathologies, including myocardial infarction (23). Furthermore, senolytic treatment improves survival and recovery in old mice following acute myocardial infarction, indicating that removal of excess senescent cells ameliorates cardiac function and prevents the manifestation of cardiac dysfunction (26). Mechanistically, the inactivation of mTORC1, a potent regulator of cellular senescence, rescues pressure overload-related heart failure and chronic myocardial infarction in mice (56, 57).

Together, these lines of evidence indicate that the prevalence of senescent cells in heart-related disorders has an important role in modulating cardiac function; however, future studies are required to establish a direct role of cellular senescence in cardiac dysfunction. Hu and colleagues recently published a comprehensive table of known markers of senescence in cardiovascular cell types and models of disease and their pathological consequences (58); however, a major limitation of studying cardiac cellular senescence is the lack of a molecular signature unique to cardiac cell types and the difficulty in assessing their impact in preclinical and clinical disease models. Thus, leveraging transcriptomic and proteomic changes in senescent cardiomyocytes would provide novel biomarkers and therapeutic targets to selectively remove or suppress senescence-related cardiac dysfunction in the future.

### Vascular Senescence

Age-related vascular dysfunction is a key antecedent to the development of clinical CVDs including atherosclerosis, coronary artery disease, peripheral artery disease, cerebrovascular disease, and aneurysms (59, 60). Vascular dysfunction is characterized by vascular endothelial dysfunction and large elastic artery stiffening (61). Vascular cell senescence has been implicated as an integrative mechanism contributing to vascular endothelial dysfunction, arterial stiffness, and CVD risk (62).

The vasculature is predominantly composed of ECs, which make up the vascular endothelium, and VSMCs, which constitute the medial layer of arteries (60). Vascular cell senescence is triggered by a variety of stressors that include replicative exhaustion, oxidative stress, oncogenic activation, telomere attrition, DNA damage, and mitochondrial dysfunction (63). Vascular tissues (i.e., the aorta) have been identified as having the greatest increase in cellular senescence burden compared with other tissues in old mice (64). This discovery suggests that vascular cells readily undergo cellular senescence because of increased exposure and/or vulnerability to cellular senescence stimulators, especially given that the vasculature is directly exposed to adverse humoral factors in circulation.

Vascular endothelial dysfunction is triggered primarily by reduced nitric oxide (NO) bioavailability which is produced in the vasculature mainly by endothelial NO synthase (eNOS) in ECs (61). Endothelial NO production is reduced with age and is further diminished in senescent ECs (65). Cellular senescence burden is associated with an increased production of ROS, which reacts with NO, reducing its bioavailability (65). Consequences of EC senescence may include dysregulation of vascular tone (66), increased endothelium permeability (67), impairment of angiogenesis (68), inhibition of vascular repair (69), and mitochondrial dysfunction (29, 70). Although EC senescence has yet to be causally implicated in endothelial dysfunction, many studies support this notion (30, 71). For example, clinical studies show that older adults exhibit greater expression of p16 and p21 (CDKN1A) in ECs and that the level of EC senescence is inversely associated with endothelial function (71). In murine models, senolytic treatment (dasatinib + quercetin or ABT-263) improved endothelial vasodilation and reduced the number of uncapped telomeres in aortic ECs (30, 72). Moreover, mounting evidence indicates that EC senescence contributes to brain aging and cognitive dysfunction, in part through cerebrovascular EC dysfunction (73). ECs isolated from microvessels in the prefrontal cortex of patients with Alzheimer’s Disease demonstrate impaired EC function and higher biomarkers of cellular senescence compared with control subjects (74). In combination, these findings suggest that EC senescence contributes to impaired endothelial function and CVD progression. More studies are required to assess the causal role of cellular senescence on endothelial function and to determine if senolytic interventions are beneficial treatments for these conditions.

Age-related arterial stiffening occurs mainly through the unfavorable remodeling of the arterial wall, which directly contributes to CVDs such as atherosclerosis and aneurysms (59). Arterial stiffness is driven by vascular oxidative stress and inflammation which amplify each other to exacerbate adverse conditions by detrimentally remodeling structural proteins in the arterial walls to induce stiffness (59, 75). VSMCs participate in arterial wall remodeling by regulating structural protein abundance and crosslinking (60, 63). Collagen deposition, elastin degradation, and crosslinking of structural proteins by advanced glycation end-products are hallmarks of arterial aging and are associated with increased senescent cell burden (61, 76). Senescent VSMCs have also been reported in atherosclerotic plaques and aneurysms suggesting a pathological role of senescent VSMCs in CVDs (77, 78). In old mice, preliminary findings indicate that senolytic treatment with ABT-263 reduced aortic stiffness and was accompanied by lower aortic intrinsic mechanical wall stiffness (72). Furthermore, senolytic treatment with dasatinib + quercetin resulted in significant reductions in senescent cell markers in the aorta from old and hypercholesterolemic mice (30). Genetic and pharmacological (ABT-263) clearance of excess senescent cells throughout different stages of atherosclerosis improved disease pathology in mouse models of atherosclerosis through ablations of LDL receptor or apolipoprotein E, providing evidence that senescent cells have deleterious effects throughout atherogenesis (7). Nonetheless, more studies are needed to explore the direct implication of vascular senescent cells in arterial stiffness and related CVDs.

Many molecular mechanisms have been implicated in vascular cell senescence. Predictions of senescence trajectories show that aortic EC senescence is associated with distinct transcription factor open chromatin states and specific mRNA expression programs (79). Despite these efforts, we have yet to uncover a vascular senescence signature, a group of genes in a cell comprising a unique pattern that is the consequence of cellular senescence, that can be widely applied to vascular aging and disease. Although the genes may change based on model system or senescence-inducing stimuli, basic criteria for a signature would include highly expressed genes in a population of cells increased by senescence induction and reduced by their removal that can be validated across either species, cell type, or induction method. Thus, developing models to study changes in transcriptomic/proteomic signature in preclinical models of vascular disease will uncover novel biomarkers and therapeutic targets to ameliorate cellular senescence-related vascular dysfunction.

## SERUM BIOMARKERS/CIRCULATING FACTORS OF SENESCENCE AND CARDIOVASCULAR DISEASE

Given the proximity of the cardiovascular system to circulation (i.e., bloodstream), both paracrine (microenvironment) and endocrine (systemic) factors may promote cardiovascular cell senescence (80). An accumulation of senescent cells originating from various tissues and organ systems alters the circulating milieu by secreting SASP factors (37). Excess SASP factors disrupt humoral factors [i.e., extracellular vesicles (EVs), immune cells, proteins, metabolites, miRNAs] in circulation leading to a dysregulated stress response and an adverse microenvironment (80). Within the cardiovascular system, SASP factors can induce systemic inflammation and oxidative stress which may directly contribute to cardiovascular dysfunction (81). Increasing evidence also supports that the accumulation of cardiovascular cell senescence is likely driven by immune cell infiltration, capillary density, and capillary recruitment in metabolically active tissues such as adipose tissues and skeletal muscle. Notably, there is increasing appreciation for the bidirectional involvement of impairments of these tissues in the pathogenesis of several CVDs such as heart failure with preserved ejection fraction (HFpEF) (12, 82–84).

SASP factors have been historically difficult to assess given the heterogeneity of the SASP profile depending on the cell type, senescent cell stage, and senescence inducer. The SASP consists of many bioactive compounds including proteins, lipids, metabolites, RNAs, and ROS. SASP factors can be secreted directly into the circulation or be packaged in EVs to be released from senescent cells. In this review, we will highlight specific SASP components known to be secreted by cardiovascular cells.

### T Cells in Cardiovascular Senescence

Peripheral blood mononuclear cells are considered both immune cells and cardiovascular cells in the right context, given their role in inflammatory responses and integration into cardiovascular tissue (85). Senescent T cells in particular have been identified as mediators of immunosenescence and as circulating markers of systemic cellular senescence (86). Several studies have identified immunosenescent CD8^+^ and CD9^+^ T cells to be specifically involved in cardiovascular dysfunction and diseases (87–89). T cells exhibit well-recognized characteristics of cellular senescence such as telomere attrition, high levels of p53 and p16, and increased SASP secretion, and are highly accessible in clinical studies (90). Thus, T cells are advantageous circulating markers of systemic cellular senescence.

### RNA in Cardiovascular Senescence

The contribution of circulating RNAs, largely comprising microRNAs (miRNAs) and long noncoding RNA (lncRNAs), to the regulation of cardiovascular function has only been recognized recently. Today, miRNAs and lncRNAs are established biomarkers and regulators of CVD, and many current studies are identifying novel circulating RNAs implicated in vascular function. miRNAs are a class of small noncoding RNAs that repress gene expression mostly by inhibiting the translation or promoting the degradation of target mRNAs with which they share partial complementarity (91). Many miRNAs have been implicated in cardiovascular cell senescence, including miR-17-92 (92), miR-494 (93), miR-217 (94), miR-34a (95) and miR-146a/b (96). In cardiovascular cells, these miRNAs are often associated with DNA damage, a hallmark, and inducer of cellular senescence, and are involved in regulating the expression of key proteins like p21 and SIRT (92, 94, 95) that function in key aging programs. LncRNAs are a vast class of RNAs lacking protein-coding capacity that have recently emerged as crucial regulators of cellular senescence (97–99). Initial studies identified lncRNA such as *NORAD*, *Meg3*, and *H19* to be involved in regulating proinflammatory and DNA damage responses, and to exacerbate atherosclerotic lesions (97–99). Together, ample evidence suggests that aging is associated with the presence of aberrant transcriptomes, including RNAs that promote cellular senescence and reduce cardiovascular function. Further studies are required to understand if dysregulated RNA causes or induces cellular senescence. Given the complexity of regulatory and compensatory pathways, further omics approaches are necessary to identify RNAs that modulate senescence networks and may represent potential therapeutic targets.

### Proteins in Cardiovascular Senescence

The composition and abundance of circulating proteins change with aging and in pathological states (100). Yet, it has been challenging to determine the cellular source of these modified secreted protein factors with aging and chronic disease. Given the recent implications of senescent cells in aging processes and various chronic disorders, it is critical to fully understand changes in circulating proteins elicited by senescent cells and the SASP (101). Well-recognized protein SASP factors include proinflammatory cytokines, chemokines, growth factors, and proteases (39). These SASP factors can induce cardiovascular dysfunction by creating proinflammatory microenvironments and degrading cardiac tissues and vascular walls (58). SASP factor analyses in senescent cardiomyocytes have identified that in contrast to the canonical SASP, the inflammatory factors IL-6 and CXCL1 were not found to be elevated, whereas noncanonical SASP factors such as EDN3 and TGF-β2, known to promote cardiac hypertrophy, were found to be upregulated, suggesting that SASP in cardiomyocytes may include not only proinflammatory cytokines but also cardiac tissue-remodeling molecules (102). SASP proteins including matrix metalloproteinases (MMP)3, MMP13, IL-1α (IL1A), and TNF contribute to arterial plaque progression by degrading structural components of the arterial wall, in turn exacerbating atherosclerosis in murine models (7). Emerging data have also revealed that the transcription factor complex NF-κB is activated by DNA damage and is a major regulator of the SASP in senescent vascular cells (103). NF-κB transcriptionally regulates the expression of many canonical SASP factors including IL-1β (IL1B), IL-6, IL-8, CXCL11, PAI1, and TNF, and inhibition of NF-κB prevents the induction of many other factors (104). Despite this evidence, other studies have shown that senescent vascular cells can also express anti-inflammatory SASP factors as a compensatory mechanism (105, 106). Because of the heterogeneity in secreted SASP factors, SASP-related databases [such as the SASP atlas (101)] have been actively updating their information to include more cell types and senescence inducers.

### Extracellular Vesicles in Cardiovascular Senescence

In addition to proinflammatory RNAs and proteins, senescent cells secrete extracellular vesicles (EVs) which play physiological and pathological roles in cardiovascular tissue (107, 108). Unlike other humoral factors in circulation, EVs may target specific cells and deliver their cargo as a means of intercellular communication (109). Senescent cells exhibit changes in EV characteristics including decreased EV size, higher EV abundance, and selective cargo molecules (110). In addition, EVs from senescent cells can induce states of cellular senescence in an endocrine manner. Cell culture studies have shown that EVs derived from conditioned media from senescent vascular cells induce cellular senescence in a paracrine manner in replicative cells and that EVs from replicative cells could reduce markers of cellular senescence when exposed to senescent vascular cells (111). Furthermore, EVs derived from senescent ECs and VSMCs have been shown to contribute to vascular calcification, a vital change leading to arterial stiffness (30). Given the targeted influence of circulating EVs on cardiovascular function, EV cargo should be further analyzed using omics-based approaches to characterize the protein and RNA contents that drive changes in cardiovascular function.

## APPROACHES TO IDENTIFY AND CHARACTERIZE SENESCENT CELLS IN CVD USING OMICS-BASED METHODS

Omics-based strategies, such as transcriptomics, proteomics, and metabolomics, investigate collectives of molecules (RNAs, proteins, metabolites, respectively) to gain a comprehensive understanding of the complexity of a biological process. These techniques enable the identification of various levels of molecular changes that occur in senescent cells in CVD and provide insights into the mechanisms driving cellular senescence and potential therapeutic targets. Furthermore, omics-based approaches can identify novel senescence markers, pathways, and cellular interactions that are difficult to detect by analyzing individual RNAs, proteins, or metabolites. New omics-based methods are being developed at a rapid pace; most recently, the advent of single-cell, spatial, and subcellular omics techniques, in addition to emerging methods for the detection of molecules in biofluids, now permit the analysis of nearly every cell type and area of tissue regardless of the model system or disease. In the following sections, we will focus on transcriptomics and proteomics, two of the most popular and accessible omics technologies that have been used extensively in the study of cardiovascular disease and aging. We will spotlight these two omics methods used to uncover changes that occur with cellular senescence, improve the identification and characterization of senescent cells, and develop strategies for therapeutic targeting and interventions for CVD.

## TRANSCRIPTOMIC STRATEGIES TO INVESTIGATE CELLULAR SENESCENCE IN CVD

Transcriptomics is the study of the complete set of RNA molecules, or transcripts, expressed in a cell, tissue, or organism (112). Transcriptomics encompasses all types of RNAs, including mRNAs, miRNAs, and lncRNAs. High-throughput sequencing methods are used in transcriptomics to analyze the expression of numerous transcripts under different conditions, often providing insight into the relationship between the transcriptome and an observed phenotype across a range of experimental entities such as cells or tissues (113).

### RNA Sequencing to Study Cardiovascular Senescence

RNA sequencing (RNA-seq) methods, developed in the mid-2000s, offer superior high throughput, lower input, and higher sensitivity than DNA microarrays (114). RNA-seq analysis is widely used as the gold standard for global analysis of RNAs and RNA isoforms; recent advances have broadened our ability to extract information on specific aspects of RNA biology, including single-cell transcriptomes, translation, RNA structure, spatial transcriptomics, and long-read sequencing to identify isoform and splicing variants (113). As RNA-seq techniques and technologies continue to advance, so will our capacity to use these methods for comprehending senescent cells in CVD.

Studying cellular senescence is complicated by the lack of consistent markers and this is magnified in animal models and human specimens with high heterogeneity of tissues and cell types. Bulk RNA sequencing, in which an entire tissue or group of cells is sequenced together, provides great depth of information but has a poor resolution for changes in expressed RNAs in a small population of cells, which is often the case for senescent cells in a given tissue. As a result, the most insightful use of bulk RNA-seq may be from primary cell cultures. In 2019, Casella and coworkers cataloged EC senescence among a variety of cell types and senescence inducers, performing RNA-seq and comparing the transcriptomic changes between each to identify common and unique gene expression programs (115). The results demonstrated that senescent gene expression is extremely heterogeneous with hundreds of differentially expressed RNAs and some RNAs shared among diverse senescent cells including human umbilical vein endothelial cells (HUVECs) (115). Uryga and colleagues used bulk RNA-seq to identify the transcriptional signatures of senescent VSMCs induced doxorubicin from male and female human donors compared with proliferating controls (116). The results revealed that human VSMC senescence has strong associations with SASP, telomere damage, DNA repair, and nuclear envelope structure. This led to further investigation and identification of persistent telomere damage as a cause of senescence and promoter of inflammation in vascular disease (116). Lastly, the genetic manipulation of cells followed by RNA-seq analysis to determine the impact of a gene of interest has also been informative, as demonstrated by Corbett et al., who discovered that ablation of FXR1 was linked to cellular senescence, reduced proliferation, and increased SASP in VSMCs (117).

Beyond cell culture, many have used bulk RNA-seq to analyze cardiovascular tissues in aging and disease. As noted above, identifying senescent cells in tissue is challenging, but not impossible. By relying on RNA-seq studies in cultured cells, we can extrapolate the data to understand patterns of gene expression reflective of senescence in tissues. For example, De Majo et al. (118) used RNA-seq analysis in combination with variant calling to demonstrate that, unlike other organs, in the mouse heart, genomic instability does not contribute to the natural process of aging and, in fact, cardiac cells maintain an active DNA repair machinery throughout life. Although this study did not specifically examine senescence, genomic instability is a classic hallmark of senescence and provided incentive to further understand the role of senescent cells in the heart. Another study used RNA-seq analysis in human blood to characterize the bone marrow stem cell response after coronary bypass surgery (119). In this example, RNA-seq analysis aided in the determination that hematopoietic stem cell (HSC) senescence likely contributes to decreased cardiac regenerative capacity by impairing the ability of the bone marrow to produce new blood cells, including those involved in repairing damaged cardiac tissue following a coronary event (119). Bulk RNA-seq was used to compare young and old mouse hearts, discovering thousands of age- and sex-dependent gene expression signatures linked to changes in exon usage and splicing patterns (120). These findings and those from Trexler et al., who also used bulk RNA-seq to identify sexually dimorphic gene expression changes in rat cardiomyocytes, could be informative as we move toward developing transcriptomic biomarkers associated with cardiovascular cell senescence (121).

In the vasculature, Childs et al. (35) applied bulk RNA-seq analysis to microdissected plaques or inner aortic arches to determine the effect of senolytic drugs on gene expression in the diseased regions of atherosclerotic mice. Sequencing results predicted that ABT-263 induced a contractile-to-promigratory VSMC switching phenotype; accordingly, the authors proposed that senescent cells inhibit VSMC phenotype switching to limit lesion entry of medial VSMCs for fibrous cap assembly (35). Similarly, Haemmig et al. (122) performed RNA-seq on the aortic intima from *Ldlr^−/−^* mice on a high-cholesterol diet during disease progression and regression stages. RNA-seq analysis revealed an evolutionarily conserved lncRNA, *SNHG12*, that was highly expressed in the endothelium during disease, and loss of this lncRNA accelerated lesion development significantly by increasing DNA damage and senescence in the vascular endothelium (122).

### Single-Cell and Single-Nuclei RNA Sequencing to Study Cardiovascular Senescence

Although bulk RNA-seq has certainly provided a new dimension to our knowledge about senescence gene expression in cells and tissues, many have since moved beyond this approach to opt for a technology known as single-cell RNA-seq (scRNA-seq), with improved cellular resolution. In 2009, Tang et al. (123) published the first study describing scRNA-seq, starting an avalanche effect that would be felt throughout the scientific community. Since then, more than 75,000 papers have been published using scRNA-seq, and many have focused on cardiovascular biology. Numerous reviews have highlighted the importance and potential applications of using scRNA-seq to exploit new therapeutic venues for combatting cardiovascular disease (124–128). Although many have chosen to use scRNA-seq over bulk RNA-seq for the improved cellular resolution, the technique may sacrifice sequencing read depth, gene diversity, or cost efficiency depending on the platform used. As a result, many have begun efforts to improve these shortcomings of scRNA-seq noted by the development of high-depth, long-read scRNA-seq. A more detailed overview of the advantages and disadvantages of scRNA-seq is reviewed for cardiovascular studies by Hegenbarth and colleagues (129).

The feasibility of scRNA-seq paired with the explosion of interest in characterizing the heterogeneity of senescent cells to improve senolytic drugs has inspired a new area of study. scRNA-seq and single-nuclei (sn) RNA-seq studies have contributed to defining the cell composition of the healthy human heart, providing transcriptional details on the common and unique cell types, and assigning transcriptional signatures to regions of the heart (130, 131). These pioneering discoveries paved the way to apply these techniques to heart pathologies as studied by Koenig et al., where snRNA-seq and scRNA-seq on heart tissue from healthy people and patients with chronic heart failure led to the finding that the levels of *TGFBI* and *NFIL3* mRNAs correlated positively with aging in dilated cardiomyopathy (DCM) cardiomyocytes (132). Previous studies have linked *NFIL3* mRNA levels to cardiac senescence and aging and TGFBI is a known component of early senescence activation and mTOR regulation in aging and cardiovascular disease (133, 134). In a study of primate aging, snRNA-seq analysis revealed that interleukin-7 (*IL7*) mRNA, encoding an SASP component, was elevated in the aged heart and that many differentially expressed RNAs in the aged heart were associated with the GenAge database, which includes senescence markers (135). In another study of primate cardiac aging, snRNA-seq analysis found that the levels of FOXP1, a core transcription factor in organ development, were reduced in aged cardiomyocytes, as were its transcriptional targets. Reductions in FOXP1 were previously implicated in the development of senescent human embryonic stem cell-derived cardiomyocytes (136).

The vasculature also represents a diverse cellular environment harboring many uses for scRNA-seq and its variations. In the aging mouse brain, scRNA-seq analysis identified 13 cell types by transcriptomic signatures, concluding that there is an increased ratio (∼10%) of senescent ECs in the cerebral microcirculation with age (137). Another benefit of single-cell techniques is the ability to multiplex the information from a given cell. Xie et al. (79) integrated scRNA-seq and single-cell analyses for transposase-accessible chromatin with sequencing (scATAC-seq) of aging mouse aortas to discover that certain endothelial subpopulations and fibroblasts are linked to open chromatin states and to subsequent matching senescent transcriptomic profiles. Other studies have exploited the benefits of scRNA-seq analysis to detect the transcriptional regulators of EC senescence, finding candidate regulators such as JUN in mice and BACH1 in humans (138, 139). snRNA-seq analysis of the aged heart revealed the dynamic state of cardiac fibroblasts, featuring a subset of fibroblasts that express antiangiogenic factors expressed by senescent cells including the serpin family of proteins (140).

Another method based on scRNA-seq analysis is cellular indexing of transcriptomes and epitopes (CITE)-seq, which concurrently quantifies cell surface protein and transcriptomic data from a single cell (141). In a recent study from our group, we used CITE-seq analysis to determine the effects of inhibiting a newly discovered senescent cell surface protein, DPP4, on cellular senescence in atherosclerosis. The technique allowed us to visualize and quantify the DPP4-positive populations of VSMCs and characterize their transcriptomes, helping us identify a group of senescent VSMCs that were selectively killed by DPP4 inhibition and another that displayed the senohemostatic state of senescent VSMCs we had uncovered in cell culture by proteomic analysis (142). These and other studies demonstrate the efficacy of scRNA-seq analysis and its many variations for identifying and characterizing senescent cells in complex model systems that require single-cell resolution.

### Spatial Resolution of Senescent Cells by In Situ RNA-Seq Analysis

So far, we have discussed the incomparable utility of scRNA-seq, and although its popularity has never been higher, many have sought to provide the spatial context within a tissue or organ to the new information gleaned from scRNA-seq. Although understanding the spatial relationship between RNAs and proteins is not new, performing such analysis in a high-throughput manner has long eluded us. Named *Nature*’s “Method of the Year” in 2020, spatially resolved transcriptomics originated from the study by Stahl et al. in 2009 (143), which first published a method for measuring the spatial distribution of transcripts by annealing fixed tissue directly to bar-coded reverse-transcriptase primers, followed by reverse transcription, sequencing, and bioinformatic analysis. Since its emergence, spatial transcriptomics has made monumental leaps, offering many commercial options for the two main techniques: spatial analysis involving transcriptomic analysis on microdissected tissues, and those using in situ hybridization, sequencing, capturing, and computational reconstruction of spatially resolved data (144). Within those techniques are a further distinction for those that measure gene expression from a region of interest harboring multiple cells, single cells, or even subcellular areas within a sample.

In recent years, cardiovascular researchers have begun implementing spatial technologies into their studies. Asp et al. (145) used spatiotemporal mapping of the developing human heart to create a publicly available cell and gene expression atlas as a new and useful resource for cardiovascular research. Spatial transcriptomics of the regenerating neonatal heart provided insightful views into the spatially and temporally regulated transcriptomic programs critical to neonatal heart repair, including a transition from proliferative to secretory phenotype of the cardiac scar and the development of a regenerative border zone by immature cardiomyocytes (146). In pathologies such as myocardial infarction and cardiomyopathy, spatial transcriptomics has been leveraged in both mouse models and human tissue, paving the way for future mechanistic studies and improved therapeutic development (147–149).

**Figure 2.**
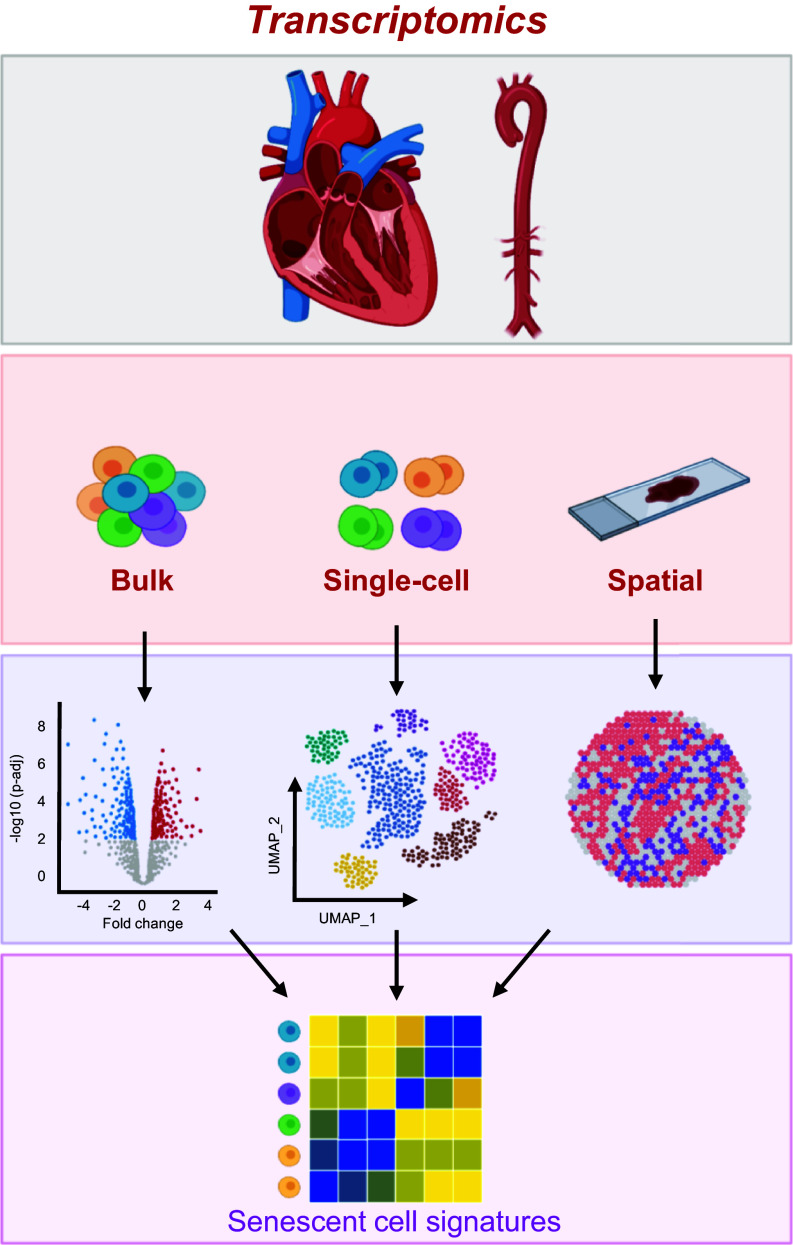
Transcriptomic approaches have emerged as powerful tools for elucidating the characteristics of senescent cardiovascular cells. The advent of RNA-sequencing technologies has significantly advanced our ability to comprehensively profile the transcriptomes of cardiac and vascular tissues in healthy, diseased, and aged states. By integrating transcriptomic data obtained from various methods such as bulk, single-cell, and spatial RNA-sequencing, we can construct highly intricate signatures specific to senescent cardiovascular cells, enabling their identification and localization in vivo. Moreover, leveraging the gene expression profiles of these cells, we can devise therapeutic strategies to combat the presence of senescent cells in cardiovascular diseases (CVDs) and aging. These transcriptomic strategies offer valuable insights into the molecular mechanisms underlying senescence-associated pathologies, facilitating the development of targeted interventions for CVDs and age-related cardiovascular complications. Images were created with a licensed version of BioRender.com and Photoshop.

Although spatial transcriptomic studies on CVD have yet to focus on senescent cells, other areas such as neurodegeneration have used spatial sequencing for this purpose. Kiss et al. (150) used spatial transcriptomics to determine that senescent cells accumulated in regions of white matter, hippocampi, and cortical gray matter in the aging mouse brain. In the human brain, spatial profiling aided in the discovery that senescent neurofibrillary tangles (NFTs) significantly differed from quiescent NFTs, appearing more similar to NFTs without tau pathology. The newly identified state of NFTs provided the framework for future studies to determine whether senescent or quiescent NFTs correlate to AD progression (151). Another study harnessed spatial transcriptomics to validate the enrichment of senescent cells in fibroblasts, ECs, and immune cells from human tissue based on comprehensive transcriptomic network analysis (152). Together, these studies demonstrate the benefit of adding spatial context to sequencing information to expand our comprehension of senescent cells and further cultivate new avenues for therapeutic intervention. In the coming years, we expect the cardiovascular field to exploit this technology in conjunction with scRNA-seq methods to investigate senescent cells in many CVDs and age-related pathologies and develop exciting new areas of intervention and prevention through the identification of senescent cell signatures (Fig. 2).

## PROTEOMIC STRATEGIES TO INVESTIGATE CELLULAR SENESCENCE IN CVD

### Emerging Proteomic Approaches for Cardiovascular Tissues

**Figure 3.**
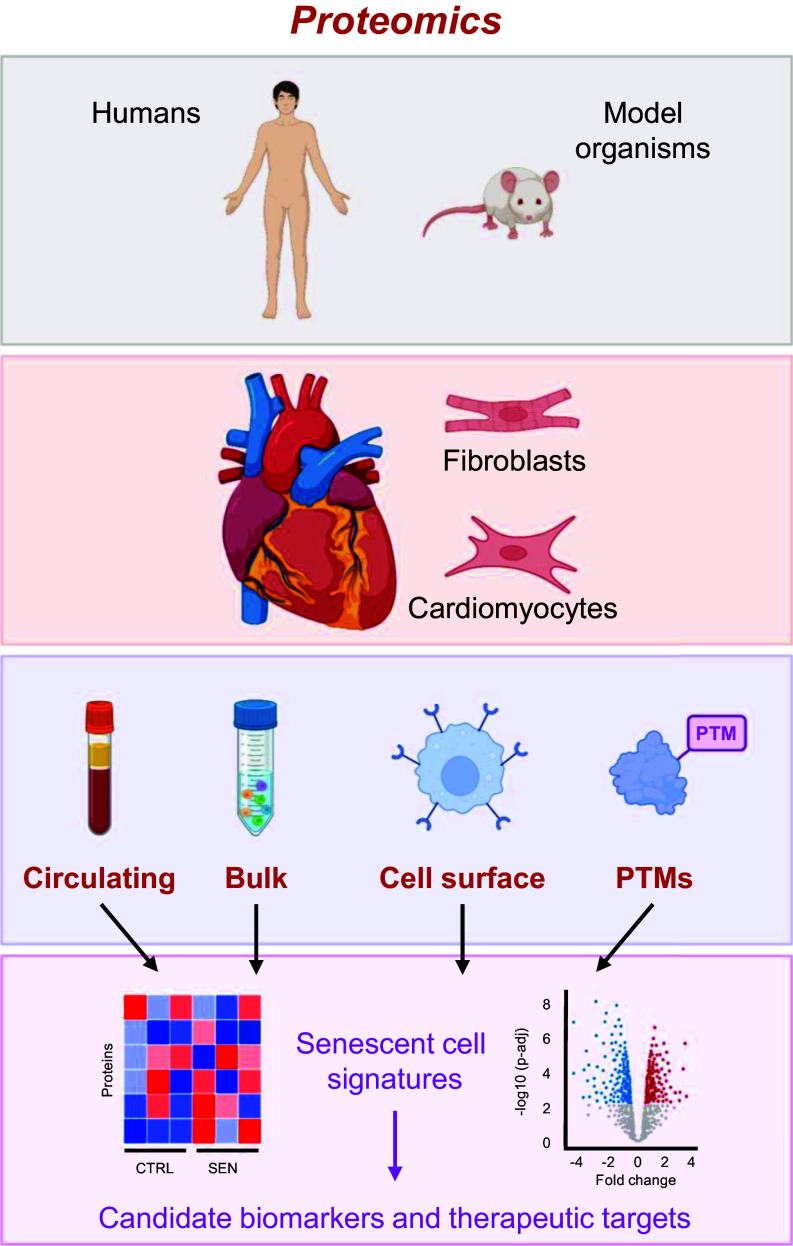
Innovative proteomic technologies for targeting and quantifying cellular senescence in the cardiovascular system. Investigation of cardiovascular senescence through proteomic analysis of in vivo models, such as human and mouse, as well as isolated cells and tissues enables novel insights into proteomic changes during cardiovascular aging. Protein alterations at different levels of analysis, including bulk proteomics, cell-surface proteomics, and post-translational modifications (PTMs), as well as the assessment of circulating proteomics from secreted factors in blood plasma or serum promote the discovery of senescence-associated molecular signatures. By combining the outcomes of proteomics strategies, we can enhance the identification of senescent cell signatures within cardiovascular tissue, leading to the discovery of potential biomarker candidates and novel therapeutic targets. Images were created with a licensed version of BioRender.com and Photoshop.

Proteomic research has long been leveraged to assess the proteome in cardiac tissues in humans and model organisms. The application of bulk proteomic approaches and mass spectrometry technologies are reviewed extensively elsewhere (153, 154). However, multiple emerging strategies that go beyond measuring bulk changes in protein abundances have proven to provide additional spatiotemporal insights that cannot be elucidated from standard untargeted proteomic approaches (Fig. 3). For example, emergence of single-cell proteomic technologies promises to revolutionize research of cardiovascular and senescence heterogeneity at cellular resolution. Although it remains a highly specialized and technically challenging method to implement, method development and dissemination of sample preparation protocols is quickly moving this field forward (155, 156) and it will eventually enter the realm of cardiovascular research. The latest single-cell proteomic approaches are capable of identifying over 2,000 proteins per cell (156).

Beyond measuring protein abundance, the use of metabolic labeling approaches (157) in combination with mass spectrometry and novel software pipelines (158, 159) has enabled the measurement of in vivo turnover rates in aging and hypertrophic hearts (160–163), as well as the reversal of cardiac aging by multiple interventions including calorie restriction, rapamycin treatment, and overexpression of mitochondria-targeted catalase. These studies have pointed to mitochondrial, metabolic, oxidative, and proteostatic mechanisms underlying the remodeling of the pressure-overloaded cardiac proteome and heart failure with preserved ejection fraction. In conjunction, proteomic analysis of human aortic biopsies revealed that aging transformed the aorta proteome by differentially regulating biological processes such as mitophagy, inflammation, and cellular senescence (164). These processes were further peturbed in aortic biopsies from patients with thoracic aortic aneurysms (164). In future studies, it will be interesting to extend protein turnover measurements in more cardiovascular tissues to uncover novel protein dynamics underlying pathologies related to cellular senescence. Furthermore, it will be of interest to extend protein turnover studies to humans, where possible, by using labeling strategies amenable to clinical studies, such as deuterium oxide, which enables metabolic labeling over the span of weeks (165).

ROS-related oxidative stress is a potent inducer and mediator of cellular senescence and is involved in atherosclerotic plaque formation. ROS bioactivity can be measured using a redox proteomic approach that assesses cysteine thiol covalent oxidative modifications. These modifications can profoundly impact protein function and are critical for understanding disease mechanisms and discovering protein biomarkers. One of the well-characterized examples of a thiol oxidation is formation of Actin Cys374 and glutathione (166), which ultimately leads to a decreased rate of actin polymerization (167). In human end-stage heart failure, there is evidence of oxidation of myofibrillar proteins, leading to impaired contractility (168). Furthermore, ischemia/reperfusion injury (I/R) in isolated rat hearts leads to an 80% increase in content of actin carbonyl groups (169). Together, these studies suggest that a detailed survey of thiol oxidation sites and their roles in cardiac function will improve our understanding of redox-dependent changes in the cardiac proteome and function. One such comprehensive survey in the heart of rats during I/R injury in the presence and absence of an antioxidant identified 4505 reversibly oxidized Cys peptides, shedding light on ROS-mediated modifications that are correlated with mitochondrial and metabolic dysfunction during the pathogenesis of I/R (170). The details of mass spectrometry approaches to assess cysteine thiol oxidation caused by ROS and applications have been reviewed elsewhere (171, 172). However, profiling cysteine thiol oxidation has technical challenges, including the low cysteine content in proteins (173) and various types of thiol oxidation modifications that occur during sample preparation. Advanced protocols have been developed (174, 175) to overcome these technical constraints and permit mass spectrometry-based methods to emerge as powerful tools to identify thiol modifications in CVD pathologies.

The cell-surface proteome (surfaceome), which is altered with aging and in senescent cells, offers a promising new directions for discovering novel biomarkers and therapeutic targets for cardiovascular aging (176). Over the past decade, various methods have emerged that enable the highly specific, quantitative, and unbiased profiling of cell surface proteins using a combination of biorthogonal chemistry to enrich the surfaceome and untargeted mass spectrometry approaches to identify such proteins. The cell surface capture (177) (CSC) approach enables the selective enrichment of surface plasma membrane proteins through the covalent modification of cell-surface proteins in living cells (in culture) with biotin, followed by streptavidin (178) enrichment at the protein or peptide levels; recent variations of this approach have greatly improved sensitivity and reproducibility by introducing automation (179, 180). Various software tools (181–183) and public databases (184, 185) now support the rigorous validation and prioritization of cell-surface protein candidates from omic studies for downstream investigation in vivo. In a major advance, a recent elegant study from Luecke, et al. leveraged μCSC and several software tools to identify 1,144 cell surface glycoproteins on cardiac cells isolated from cryopreserved human hearts. Among these, LSMEM2 was a novel cell-surface protein specific to healthy cardiomyocytes and was one of many cell-surface proteins differentially abundant in healthy versus failing cardiomyocytes. This study demonstrated the utility of cell-surface proteins for identifying and targeting aged cardiac cells. Several research groups have also shown the utility of identifying and targeting proteins on the senescent cell’s surface (176, 186). Notably, DPP4 is enriched on the senescent cell surface and has been identified as a therapeutic target for improving plaque stability in the vasculature of mice (142). However, the study of the senescent surfaceome is in its early days. Due to the heterogeneity of senescent cells depending on cell type, cellular senescence-inducing stimuli, and biological context, future studies that rigorously profile the senescent cell surface in multiple cell types that exist in the vasculature will be essential for the identification of senescent cell populations in cardiovascular tissues and the development of future cardiovascular-specific senotherapuetics.

### Technologies for Serum and Plasma Protein Biomarker Discovery for Cardiovascular Aging and Disease

Blood-based biomarkers are powerful tools for clinical research and are promising sources of biomarkers to quantify age-related declines, senescent cell burden, and CVD risk (187–189). The discovery of blood-based biomarkers of senescent cells began with the discovery of the SASP, a rich source of potentially secreted biomarkers, initially profiled using cytokine arrays (3). These early proteomic studies described some of the best-known inflammatory SASP components such as IL-6, IL-8, IL-1β, and multiple growth factors. Later the SASP was studied with more comprehensive spectrometry-based proteomic approaches that generated an unbiased survey of the SASP, revealing a much more robust phenotype consisting of hundreds of protein changes that are heterogeneous depending on cell type and senescence-inducing stimuli (101). In cell culture, one may generate pure and relatively homogenous populations of senescent cells under defined conditions using one of multiple senescence-inducing stimuli. The conditioned medium of cultured senescent cells can be used for SASP profiling using untargeted mass spectrometry-based approaches, a protocol detailed elsewhere (190, 191). In addition to a soluble SASP, senescent cells secrete bioactive extracellular vesicles (EVs) containing a distinct proteomic profile (110, 191) that can also serve as an expanded and novel set of candidate biomarkers that remain to be comprehensively assessed in human studies. Methods for the isolation and comprehensive assessment of a purely isolated EV-SASP are under development (192).

Numerous studies have leveraged a biomarker discovery pipeline (187) that begins with the development of candidate senescence biomarkers in cell culture studies using one of several available proteomic approaches and moves onto validation of biomarker candidates in human plasma studies. Early studies in human plasma suggested the presence of senescence-associated proteins in circulation are biomarkers for multiple clinical outcomes associated with cellular senescence, including aging, medical risk, and associated clinical outcomes: mortality, multimorbidity, mobility, and menopause (100, 101, 193–196). Preclinical studies have identified several putative SASP factors that influence arterial function (28). SASP proteins associated with arterial stiffness were primarily related to metabolic processes (e.g., glycolysis, gluconeogenesis), amino acid biosynthesis, and hypoxia-related signaling, whereas SASP influencing endothelial function include NO altering processes such as biosynthesis, glycoprotein synthesis/degradation, cell adhesion, glutathione metabolism (e.g., oxidative stress), and PI3K-Akt signaling (28). Other preclinical studies have also identified pathological levels of cellular senescence-associated factors in the blood as drivers of changes in hemostasis and contributors to atherosclerosis (142, 197). Due to their proximity to the circulating milieu, the heart and vascular tissues are likely to secrete factors into the blood that could be leveraged as biomarkers. Translation of therapies that target senescent cells, such as senolytics and senomorphics, into the clinic will require biomarkers of cellular senescence levels to identify and stratify individuals with elevated senescent cell burden, who will presumably most benefit from the removal of excess senescent cells. In addition, plasma biomarkers will verify the efficacy of the intervention following the removal of senescent cells. Thus, the emergence of novel technologies and workflows for deep interrogation of the blood proteome is key to developing cellular senescence and CVD biomarkers.

The untargeted discovery of protein biomarkers in serum and plasma is a technical challenge due to the large dynamic range of protein concentrations in plasma, exceeding 10 orders of magnitude (198, 199). Albumin alone usually comprises more than half of the total protein, and the 12 most abundant components comprise over 95% of the total protein (200). Because of these challenges, large-scale proteomic methods that have long focused on blood are severely limited to detecting only the most abundant circulating proteins, which are not necessarily the most relevant proteins or biomarkers. Unless coupled with one of several available strategies for reducing the dynamic range of protein samples, traditional methods of plasma proteomics are limited. Here we provide a brief overview of the strategies used to discover serum and plasma biomarkers.

A common strategy to reduce the dynamic range of proteins in blood samples is abundant protein depletion. Abundant protein depletion with columns containing immobilized antibodies for up to 20 proteins is commonly incorporated into mass spectrometry-based proteomic workflows of plasma and serum, resulting in gains of proteome depth up to 25% with relatively little added effort (201). Another common strategy to reduce dynamic range is extensive fractionation of plasma using one of many available methods for chromatographic peptide and protein fractionation. A combination of deep fractionation (i.e., 30 fractions) and abundant protein depletion have been used to achieve depths of >4,500 proteins in plasma with low technical variability (202). Isobaric tagging, fractionation, and abundant protein depletion methods can be used alone or in combination to improve the depth and throughput of biomarker studies, as illustrated in the study by Kristensen et al., who identified 712 proteins in a plasma proteomic study of atherosclerosis (203). Vinculin was the most elevated protein in CVD, along with multiple other biomarkers such as serum amyloid A and C reactive protein (203).

The development of mass spectrometry acquisition strategies and analysis pipelines has immensely improved data reproducibility, quantification, and completeness in mass spectrometry workflows. In particular, data-independent acquisition (DIA or SWATH) methods have been demonstrated to be highly reproducible and quantitative in large-scale studies (204). These workflows are ideal for biomarker studies due to their superior performance relative to traditional data-dependent analysis (DDA) approaches but with reduced stochasticity of protein identifications, resulting in fewer missing data points between samples (205). DIA methods also improve quantitative dynamic range over label-free DDA methods (206) and, along with other mass spectrometry analyses, are increasingly used in clinical biomarker research (207, 208). For example, one application of DIA/SWATH in human coronary arteries and aortas identified hundreds of proteins that are associated with early atherosclerosis and a 13-protein panel that was predictive of coronary artery disease in an independent clinical cohort (AUC = 0.92) (209). Given its success in clinical plasma proteomic studies applied in scenarios such as COVID-19 severity (210), DIA/SWATH remains a promising method for the discovery and validation of plasma biomarkers in the context of heart disease. The recently developed nanoparticle-based method has also helped to overcome some of the aforementioned limitations of protein biomarker discovery in plasma and serum (211, 212). Nanoparticles can reduce the proportion of highly abundant proteins and enrich low-abundance proteins in serum and plasma. Notably, the composition of “protein coronas,” proteins at the interface of the surface of nanoparticles and the biological sample, can be tuned for swaths of plasma proteins in a reproducible manner, enabling the use of nanoparticle-based preparation workflows in combination with mass spectrometry analysis for scalable biomarker studies with superior protein depth exceeding 2,000 proteins (213). Although this technology has yet to be applied in the context of cardiovascular disease research, it promises to greatly expand coverage of the circulating protein biomarkers in future studies.

Recently, nonmass spectrometry-based technologies have emerged with the capability to reproducibly measure thousands of proteins in human biofluids. The SomaScan assay uses libraries of slow-off rate-modified aptamers (SOMAmers) with chemically modified nucleotides for affinity enrichment of proteins from complex samples. Plasma or serum proteins are measured indirectly by transforming protein concentrations into a pattern of DNA aptamer levels, which is ultimately quantified on a DNA microarray (214, 215). Recently, the SomaScan was used to generate a healthy aging signature, identifying 217 age-associated proteins from plasma, while another SomaScan study discovered a plasma proteomic clock to predict age (100, 195, 216). In a notable study of cardiovascular risk biomarkers from 32,130 archived plasma samples across nine clinical studies, the SomaScan 5,000 protein panel was used to develop a machine learning-based 27-protein panel that was predictive of myocardial infarction, stroke, heart failure, and death (217). The latest SomaScan assay provides reproducible measurements for 7,000 proteins from a single plasma or serum sample (218). Another technology recently developed, the proximity extension assay (PEA), detects proteins using antibody pairs conjugated to oligonucleotides. Upon successful binding of the protein by both antibodies, the oligonucleotide probes anneal and can be extended by a DNA polymerase and quantified by real-time polymerase chain reaction, thereby increasing the detection sensitivity and specificity (219). A PEA-based panel of 157 cardiovascular and inflammatory biomarkers was trained and validated in multiple coronary heart disease patient cohorts to identify 18 biomarkers that are predictive of cardiovascular-associated death (220).

In combination with transcriptomics and other forms of proteomics, we anticipate the use of various emerging proteomic technologies for identifying circulating protein biomarkers of senescence and CVD will greatly increase in frequency and will be highly effective and valuable as more cardiovascular-related studies focus on this important area of diagnostic discovery.

## CONCLUSIONS AND PERSPECTIVES

Senescent cells are abundant and harmful in age-related pathologies, particularly in CVDs and age-related cardiovascular functional decline. The field of cellular senescence has made great efforts to characterize these cells in different tissues, diseases, and ages, aided significantly by the emergence of omics technologies. Although significant knowledge has been gained about senescent cells in cardiovascular cells and preclinical and clinical models of CVD and aging, we have not yet found a therapeutic solution to combat the contribution of senescent cells to cardiovascular decline and disease. The study of cellular senescence in cardiovascular cells and tissues has undergone tremendous development, from investigating cells in culture with bulk RNA-seq and proteomics to in vivo spatiotemporal transcriptomic and proteomic methods, such as scRNA-seq, which has helped to uncover unique senescent cell populations and their characteristics or functions within a tissue. However, the need for combining and leveraging omics-based approaches in cardiovascular research is growing.

Several genomic technologies, such as single-cell multiomics (RNAseq + ATACseq), CITE-seq, and spatial transcriptomic profiling, now exist. Moreover, multiple technologies and workflows are now available in the proteomic arsenal to profile in vivo proteome dynamics, senescent biomarkers, and potential therapeutic targets, including metabolic labeling and mass spectrometry for proteome turnover, cell-surface capture workflows, and multiple emerging approaches increase the depth, throughput, and sensitivity of biomarker discovery in plasma and serum samples. The next phase of investigating senescent cells in CVD is using these techniques on more informative samples and populations, such as human clinical samples and complex preclinical models of CVD and cardiovascular aging, to capture the dynamic state of cellular interactions and communication. Furthermore, we can continue to push the boundaries of omics approaches by incorporating live-cell omics, RNA variation sequencing (such as long-read sequencing of spatial profiling to capture isoform and splicing variants), and 3-D reconstruction of spatial omics, among other potential future directions. Although this review focused on transcriptomic and proteomic strategies, other scalable omics include genomics, metabolomics, epigenomics, and lipidomics; multiplexing these technologies will permit the acquisition of critical information about cardiovascular tissue. It will also be important to generate more sophisticated preclinical model systems of CVD and cardiovascular aging, including cellular senescence reporter systems that are tissue-specific, lineage-tracing animals for senescent cells from a given cell type or tissue, and animals that can provide information on the cells that secrete specific proteins with detrimental systemic effects. We can pair these improvements with increasingly complex forms of computational analysis, including analysis of cellular networks and neighborhoods, communication, and signaling, and machine learning-generated models to predict disease development based on gene expression and spatial information.

We expect that future use of omics-based approaches at increasingly high resolution and detail will provide valuable insights to develop therapeutic strategies to intervene or delay the onset of cardiovascular aging and age-related CVDs. To ensure these efforts are worthwhile, we must continue to be rigorous in validating these findings by testing them in multiple experimental models or systems and using several forms of confirmation before moving forward with therapeutic solutions. In addition, we should pair the omics-based discoveries with physiological and functional changes that are measurable and meaningful. We anticipate the next decade will see major breakthroughs in developing novel therapeutics gained from omics studies focused on senescent cells in CVD and cardiovascular aging.

## GRANTS

This work was supported by Longevity Impetus grants (to N.B.) and a SenNet National Institutes of Health Common Fund Grant through National Institute of Aging Grant U54 AG079779 (to N.B.; Dr. Jennifer Elisseeff, principal investigator). 

## DISCLOSURES

No conflicts of interest, financial or otherwise, are declared by the authors.

## AUTHOR CONTRIBUTIONS

S.A.M., A.K.D., N.B., and A.B.H. conceived and designed research; S.A.M. and A.B.H. performed experiments; S.A.M. and A.B.H. analyzed data; S.A.M. and A.B.H. interpreted results of experiments; S.A.M., A.K.D., and A.B.H. prepared figures; S.A.M., A.K.D., N.B., and A.B.H. drafted manuscript; S.A.M., A.K.D., N.B., and A.B.H. edited and revised manuscript; S.A.M., A.K.D., N.B., and A.B.H. approved final version of manuscript.
